# Cryoglobulinemia: An update on classification, pathophysiology, clinical presentation, and management

**DOI:** 10.1111/joim.70042

**Published:** 2025-11-26

**Authors:** Anna Linda Zignego, Laura Gragnani, Marcella Visentini, Riccardo Bomben, Luca Arcaini, Clodoveo Ferri, Valter Gattei, Cesare Mazzaro

**Affiliations:** ^1^ Department of Experimental and Clinical Medicine University of Florence Florence Italy; ^2^ Department of Translational Research and New Surgical and Medical Technologies University of Pisa Pisa Italy; ^3^ Department of Translational and Precision Medicine Sapienza University of Rome Laboratory affiliated to Istituto Pasteur Italia‐Fondazione Cenci Bolognetti Rome Italy; ^4^ Clinical and Experimental Onco‐Hematology Unit Centro di Riferimento Oncologico di Aviano (CRO) IRCCS Aviano Pordenone Italy; ^5^ Department of Molecular Medicine University of Pavia Pavia Italy; ^6^ Division of Hematology Fondazione IRCCS Policlinico San Matteo Pavia Italy; ^7^ Department of Medical and Surgical Sciences for Children and Adults University Hospital of Modena and Reggio Emilia Modena Italy

**Keywords:** autoimmune disorders, Cryoglobulinemia Type I, hepatitis B virus, hepatitis C virus, lymphoproliferative disorders, Type II and Type III mixed cryoglobulinemia

## Abstract

Cryoglobulinemia (CG) is defined by the presence of serum immunoglobulins that precipitate below 37°C and redissolve upon rewarming. It is classified into three types based on immunoglobulin composition. Type I, a rare form, involves monoclonal IgM or IgG and is linked to lymphoproliferative disorders. Types II and III are known as mixed CG (MC): Type II consists of polyclonal IgG and monoclonal IgM with rheumatoid factor (RF) activity, whereas Type III includes polyclonal IgG and polyclonal IgM with RF activity. MC is predominantly associated with hepatitis C virus (HCV) infection and involves B‐cell lymphoproliferation and autoantibody production. CG may lead to systemic vasculopathy, ranging from mild symptoms (purpura, asthenia, and arthralgia) to severe complications such as skin ulcers, peripheral neuropathy, renal involvement, and non‐Hodgkin lymphoma. Compared to MC, Type I is more often marked by severe cutaneous involvement (ulcers, gangrene), hyperviscosity, and a higher risk of morbidity due to the underlying hematologic malignancy. Management of Type I requires control of vasculopathy and treatment of the hematologic neoplasm, whereas MC demands antiviral therapy in all HCV‐associated or hepatitis B virus–associated cases. Severe vasculopathy in both types may benefit from corticosteroids, immunomodulators, anti‐CD20 monoclonal antibodies, and plasma exchange. A multidisciplinary approach is essential for addressing both etiology and complications, thereby improving outcomes. This review summarizes the pathophysiology, clinical features, recent etiopathogenetic insights, and therapeutic advances related to the various forms of CG.

## Graphical abstract

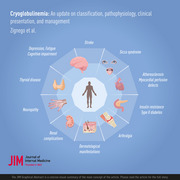

## Classification of cryoglobulinemia

Cryoglobulinemia (CG) refers to the presence of circulating immunoglobulins in the serum that precipitate at cold temperatures (<37°C) and redissolve upon rewarming. According to Brouet et al. [1], CG is classified into three subgroups based on immunoglobulin composition (Table 1): Type I CG consists of a single monoclonal immunoglobulin isotype, typically IgM or IgG, and rarely IgA. Type II and Type III are defined as mixed CG (MC): Type II MC consists of monoclonal IgM and polyclonal IgG with rheumatoid factor (RF) activity, and Type III MC consists of both polyclonal IgG and IgM, also with RF activity [2].

**Table 1:** Classification of cryoglobulinemia.

Type of cryoglobulinemia	Composition of immunoglobulins	Main association or underlying diseases	Prevalence (%)
Type I	Single monoclonal Ig (usually IgM, IgG)	Lymphoproliferative disorders: MGUS, WM, MM, NHL, CLL	5–10
Type II	Monoclonal component (usually IgM) and polyclonal (usually IgG) RF activity	**Secondary to infection**: HCV (80%–90%) other infection (HBV, HIV) **Secondary non‐infection**: Auto‐immune disease (SS, RA, SLE, B‐NHL) **Essential** (1%–13%)	50–65
Type III	Both policlonal component (usually IgM and IgG) RF activity	**Secondary to HCV** (80%–90%): other infection (HBV, HIV) **Secondary non‐infection**: Autoimmune disease (SS, RA, SLE, B‐NHL) **Essential** (1%–13%)	30–40

Abbreviations: MGUS, monoclonal gammopathies of uncertain significance; MM, multiple myeloma; RA, rheumatoid arthritis; RF, rheumatoid factor; SLE, systemic lupus erythematosus; SS, Sjögren syndrome; WM, Waldenström's macroglobulinemia; NHL, non‐Hodgkin lymphoma; B‐NHL, B‐cell non‐Hodgkin lymphoma; CLL, chronic lymphocytic leukemia.

### Type I cryoglobulinemia (CG)

Type I CG is almost always associated with lymphoproliferative disorders (LPDs), whether indolent, smoldering, or malignant. These include monoclonal gammopathy of undetermined significance (MGUS), Waldenström's macroglobulinemia (WM), multiple myeloma (MM), non‐Hodgkin lymphoma (NHL), and chronic lymphocytic leukemia (CLL) [1, 3, 4, 5]. Type I CG is only rarely associated with autoimmune diseases (Sjögren syndrome—SS, rheumatoid arthritis—RA, systemic lupus erythematosus—SLE), more typically in the presence of mixed or overlapping forms.

Type I CG with IgM monoclonal protein (M‐protein) is distinct from the IgG isotype in that it arises in the context of underlying WM, other NHL, or an IgM MGUS, as opposed to Type II and Type III mixed cryoglobulins, which are typically associated with viral infections and autoimmune diseases (see also below) [6]. Symptomatic CG with IgM MGUS falls under the category of IgM monoclonal gammopathy of clinical significance (MGCS), which includes cold agglutinin disease, anti‐MAG neuropathy, Schnitzler's syndrome, and immunoglobulin light chain amyloidosis, which may coexist. Often, bone marrow infiltration is sparse (<10%), but the circulating IgM M‐protein behaves in a highly pathogenic manner [7, 8].

### Type II and Type III cryoglobulinemia (MC)

In Type II MC, there is a monoclonal component possessing avidity for the polyclonal component of a different isotype (most frequently IgM with RF activity: an autoantibody directed against the Fc portion of IgG). Type III MC consists of both polyclonal IgG and IgM, also with RF activity. The RF detected in Type II CG in most cases is a monoclonal IgMk.

MC is strongly associated with chronic hepatitis C virus (HCV) infection in 80%–90% of cases [9]. It can also occur in 1%–4% of cases due to chronic hepatitis B virus (HBV) infection, B‐cell LPDs, and autoimmune diseases, including SS, SLE, and RA [5, 10]. In 5%–10% of cases, MC remains idiopathic and is classified as essential CG [3, 5, 11] (Table 1).

## Pathophysiology

CG can lead to tissue damage through two main mechanisms: the aggregation of cryoglobulins in the microcirculation and the immune complex‐mediated inflammation of blood vessels and surrounding tissue. This latter condition is known as cryoglobulinemic vasculitis (CV) and predominantly affects capillaries, venules, or arterioles [12, 13].

When cryoglobulins aggregate, they obstruct blood flow to joints, muscles, and organs. In Type I CG, a key pathophysiologic role is played by Type I agglomerates in the context of elevated cryocrits as mechanical factors determining the onset of ischemic symptoms. Micro‐thromboses typically cause multiple thromboses in small‐ and medium‐sized vessels and, less frequently, vascular inflammation. Type I cryoglobulins rarely exhibit RF activity, so complement‐mediated inflammatory vasculitis is less common in Type I CG than in MC [3, 5, 11, 14]. Notably, Monoclonal IgG cryoglobulins form organized networks that trap cells in blood vessels—a phenomenon known as rouleaux formation [15]. Regarding the renal disorders observed in the course of Type I CG, laboratory animal experiments have suggested that immune complex deposition in glomerular capillaries is the primary pathogenic mechanism, rather than complement or Fc receptor‐mediated nephrotoxicity [16, 17].

By contrast, Type II and Type III MCs are true inflammatory small‐vessel vasculitides driven by complement‐mediated immune complex deposition. In these conditions, IgM with RF activity forms complexes with IgG and complement, leading to tissue inflammation and injury [14].

Although the two types of CG (Type I CG and MC) are considered entirely different conditions, Type I CG is typically associated at presentation with LPDs, and MC is considered a pre‐lymphomatous LPD. In fact, it is by far the most significant risk factor for B‐cell lymphoma in patients with chronic HCV infection, conferring a risk 35 times higher than in the general population [18]. The most common HCV‐related LPDs in this context are marginal zone lymphoma (MZL), lymphoplasmacytic lymphoma (LPL), and high‐grade diffuse large B‐cell lymphoma (DLBCL) [19].

## Clinical manifestations of cryoglobulinemia (including both type I and MC)

**Fig. 1:**
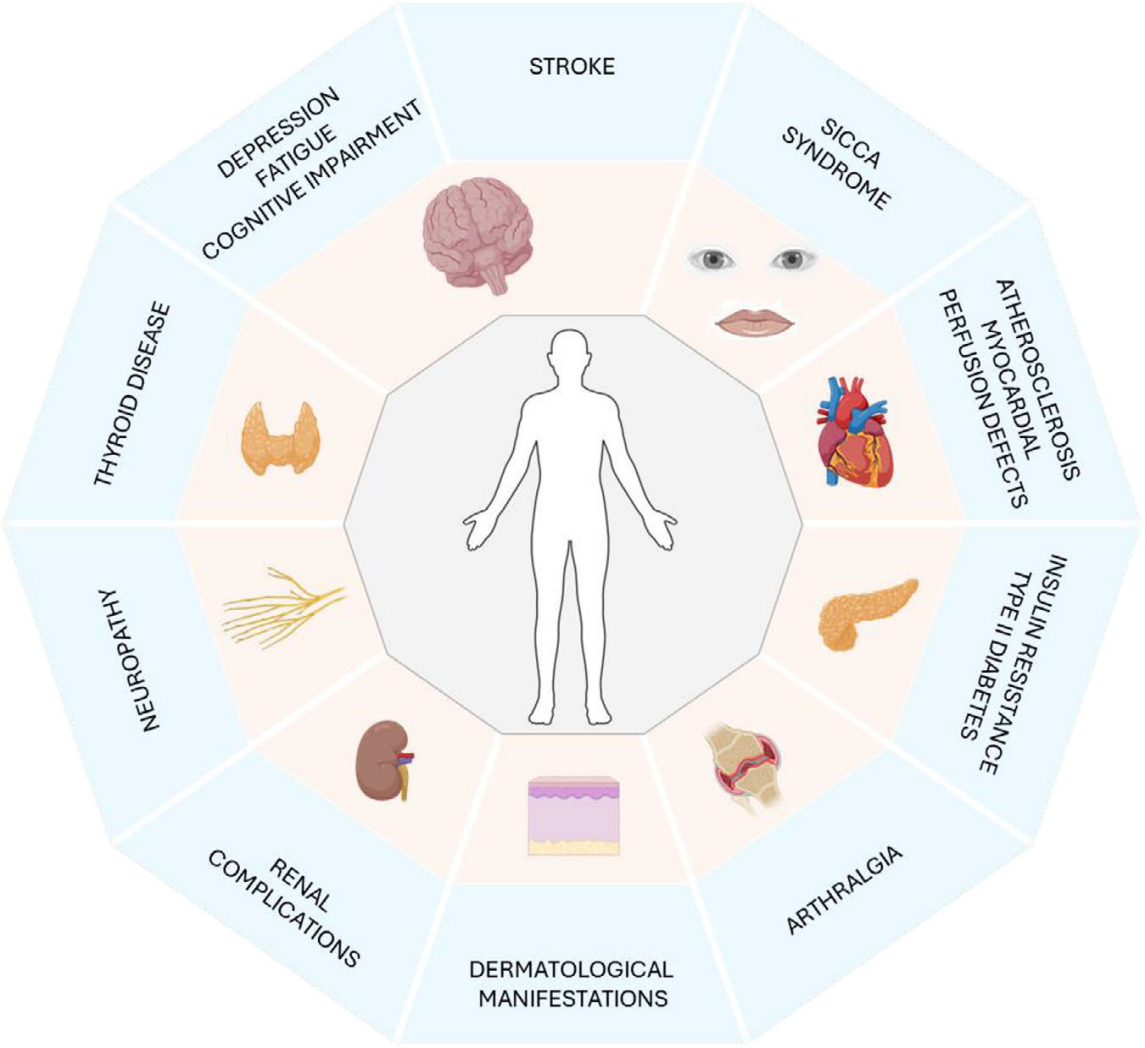
The clinical manifestations of cryoglobulinemia are highly diverse and may affect nearly every organ, thereby classifying it as a systemic disease.

The clinical manifestations of CG are highly diverse and may affect nearly every organ, thereby classifying it as a systemic disease. Fig. 1 and Table 2 show the main symptoms that may be observed in the course of CG [14].

**Table 2:** Main cryoglobulinemia symptoms.

Affected tissue or organ	Clinical manifestations
Cutaneous	Purpura, livedo reticularis, cold urticarial, acrocyanosis, distal ischemia, ulcers, necrosis
Neurological	Neuropathy (peripheral, rare central)
Renal	Glomerulonephritis, nephrotic syndrome
Hyperviscosity	Bleeding, visual disturbance, headache, CNS ischemia
Musculoskeletal	Myalgia, arthralgia, arthritis
Gastrointestinal/Pulmonary/Cardiac	Rare (MC): abdominal pain, nausea, vomiting, and in severe cases, bowel ischemia or bleeding; cough, dyspnea, or hemoptysis; clinical picture of myocarditis, pericarditis, or even heart failure
Red flag features	symptomatic hyperviscosity, critical ischemia, rapidly progressive renal impairment or neuropathy

Abbreviations: CNS, central nervous system; MC, mixed cryoglobulinemia.

Both Type I CG and MC can be asymptomatic, with clinical presentations ranging from asymptomatic cases to those with typical symptoms despite undetectable cryoglobulins [5, 20]. Indeed, the presence of circulating cryoglobulins is the primary biomarker used to diagnose CG; however, some patients exhibit classic symptoms even in the absence of detectable cryoglobulins [3, 5, 21]. This situation may result from multiple, often concomitant factors. These include, first of all, the inherent challenges in the accurate detection of cryoglobulins in routine laboratory practice, which requires strict pre‐analytical conditions both for the determination of cryoglobulins present in serum (with the consequence of possible false‐negative results) [22] and to prevent ex vivo cryoprecipitation. In addition, certain conditions may act as confounders during diagnostic testing, including the presence of cryofibrinogen, particularly relevant in Type I CG [14]. Furthermore, there is considerable temporal variability in cryoglobulin levels, which may fluctuate below the threshold of laboratory detection, even though small amounts of measurable cryoglobulins can still result in clinically significant symptoms [6]. Repeat testing is recommended in cases of clinical suspicion, and laboratory results should always be interpreted carefully in the context of the clinical picture by a properly trained specialist with experience in the diagnosis of CG [23].

Additionally, assessing RF levels (high) status and the C4 levels (low), as well as the presence of hypergammaglobulinemia (often with a monoclonal spike), can be helpful. If the diagnosis remains uncertain, a biopsy of affected tissue (e.g., skin, kidney, or peripheral nerve) may yield a definitive diagnosis [14, 20, 22].

In clinically overt cases, manifestations range from mild symptoms (e.g., leg purpura, arthralgia) to severe or even catastrophic clinical presentations, as will be detailed in the following paragraphs, which summarize the main clinical features described in the literature for the different types of CG. In any case, to ensure an appropriate clinical and therapeutic approach, it is essential to first consider the potential presence of “red flag features,” such as symptomatic hyperviscosity, critical ischemia, rapidly progressive renal impairment, or rapidly evolving neuropathy.

### Clinical manifestations of Type I CG

The typical symptomatology of Type I CG (Table 3) is largely derived from retrospective studies [21, 23, 24, 25]. This condition is characterized by more severe cutaneous manifestations and hyperviscosity syndrome, due to the high concentration of cryoglobulins. Skin manifestations are highly dependent on external cold and mostly involve extremities’ hypoperfusion (e.g., acrocyanosis or Raynaud's phenomenon). Cutaneous necrosis is much more marked than in MC. Hyperviscosity symptoms include mucosal bleeding and neurosensory signs, encompassing blurred or diminished vision, diplopia, headache, confusion, deafness, vertigo, nystagmus, ataxia, stroke, or even coma. The cerebral involvement, very rare in MC, is typical of Type I. Arthralgias occur less frequently than in MC [14]. Renal involvement in both Type I and MC is clinically similar, often presenting as nephrotic proteinuria, hypertension, hematuria, and renal failure. In Type I CG, kidney damage may also stem from the underlying hematologic disorder, such as myeloma‐related nephropathy. The course of disease is variable, and prognosis is primarily influenced by the extent of the related underlying hematologic malignancy and/or vasculopathy‐related multiorgan involvement. In severe cases, it is considered life‐threatening [26].

**Table 3:** The most common clinical manifestations observed in Type I cryoglobulinemia (CG).

Most frequent features of Type I cryoglobulinemia
Acrocyanosis (blueness of the hands and feet from the cold)
Raynaud phenomenon
Gangrene
Arterial thrombosis
Hyperviscosity syndrome

In a recent national multicenter study conducted in France on 168 patients with Type I CG, patients with IgG isotype exhibited more severe manifestations (greater renal involvement and cutaneous necrosis), more relapses, and lower survival compared to those with IgM. Renal involvement was identified as an independent negative prognostic factor. The primary causes of death were infections, hematologic disorders, and heart failure [23]. Among the factors independently associated with poor event‐free survival are the IgG isotype and kidney involvement. In a very recent study involving over 500 patients with monoclonal IgM disorders, nearly one quarter had Type I CG, and more than half of these were symptomatic. The most prominent symptoms were cutaneous manifestations, vasomotor disturbances, and hyperviscosity syndrome. Notably, patients with underlying MGUS, and consequently lower concentrations of monoclonal protein, were more likely to be symptomatic (MGCS) [6].

### Clinical manifestations of Type II and Type III MC (Table 4)

#### HCV‐related MC (HCV MC)

Data on MC primarily comes from cases of HCV‐related MC (HCV MC). In fact, after HCV identification, it was established that 90%–95% of previously termed “essential MC” cases were HCV‐associated [9, 27, 28, 29], and MC became recognized as a prototype of HCV extrahepatic manifestations (EHMs‐HCV) [30, 31, 32]. MC is a complex disorder that mimics most EHMs‐HCV [30, 33], with chronic HCV infection triggering autoreactive and lymphoproliferative processes [34, 35]. In fact, HCV infects both hepatic and lymphoid cells, leading to B‐cell lymphoproliferation and immune complex/cryoglobulin formation, which drive vasculitic symptoms (see below).

HCV MC presents with varying severity [5, 12]. Mild‐to‐moderate symptoms include purpura, fatigue, arthralgia, mild neuropathy, and glomerulonephritis with preserved renal function, whereas severe cases can develop extensive skin disease, severe neuropathy, renal impairment, and gastrointestinal involvement. Life‐threatening complications include rapidly progressive glomerulonephritis, central nervous system (CNS) involvement, intestinal ischemia, and alveolar hemorrhage [26, 36]. Patients range from asymptomatic (25%–30%) to severe cases (2%–5%).

Skin involvement is characterized by gravity‐dependent vascular purpura, livedo reticularis, subcutaneous nodules, bullae, vesicles, and cold urticaria, often triggered by physical exertion or prolonged standing [14, 36, 37, 38].

Neurologic involvement most commonly manifests as sensorimotor polyneuropathy, starting in the feet and rarely extending above the knees. It may also involve the hands, though typically not beyond the wrists. Sensory symptoms often precede motor deficits by months or years. Less commonly, multiple mononeuropathies mimic periarteritis nodosa, whereas rare cases of small‐fiber neuropathy cause widespread pain. CNS involvement is rare but may present with focal deficits or neurocognitive disorders [14, 36]. Joint and muscle involvement is common—presenting as inflammatory, bilateral, symmetric, and nondestructive arthralgias of large joints—whereas synovitis is rare. MC can mimic RA, but the absence of bone erosions and anti‐citrullinated protein antibodies in most cases helps distinguish it [2, 14]. Renal involvement occurs in about one third of patients, typically as membranoproliferative glomerulonephritis (MPGN) with proteinuria, hypertension, hematuria, and renal failure. Renal biopsy reveals three main patterns. About 80% of patients have a diffuse MPGN. Glomerular basement membrane thickening with double contour appearance, endocapillary proliferation with mesangial enlargement and intracapillary accumulation of neutrophils and monoclonal cells, especially monocytes, and luminal obstruction by cryoglobulin (the so‐called pseudo‐thrombi) are the main histological features. Crescents and necrosis are relatively uncommon. Lesions are more often diffuse. In 10% of patients, the MPGN is focal. About 10% of patients have mesangial proliferative glomerulonephritis. Immunofluorescence stains are positive for IgM (usually IgMk). Strong IgM and IgG deposition is observed in the thrombi, whereas fibrinogens can be detected in the vessel walls. Relapses may lead to fibrosis, but end‐stage renal disease is rare [3, 39]. Systemic symptoms—including fatigue and, less frequently, fever—may be observed during disease relapses. MC may also be associated with sicca syndrome (in most cases seronegative for SS‐A/SS‐B antibodies) and Raynaud's phenomenon [40, 41]. Other vasculitic complications are rare and can affect multiple organs. Gastrointestinal involvement may result from mesenteric vasculitis, leading to abdominal pain, nausea, vomiting, bowel ischemia, or bleeding [42, 43]. Pulmonary involvement may be presented as alveolar hemorrhage, interstitial lung disease, or pulmonary capillaritis, with symptoms, such as cough, dyspnea, or hemoptysis in severe cases [44]. Cardiac involvement is infrequent but may include myocarditis, pericarditis, or heart failure. Coronary vasculitis can cause myocardial ischemia or arrhythmia. HCV MC has also been linked to malignant B‐cell LPDs, including MZL, LPL, and aggressive DLBCL [30, 45, 46, 47]. In addition to the common associations, thyroiditis and thyroid disorders have also been reported in patients with HCV MC, with a higher incidence of autoimmune thyroid disease, as shown in a longitudinal study [48]. Moreover, although less frequent, there is evidence suggesting that insulin resistance, diabetes, and atherosclerosis may also be associated with HCV infection, contributing indirectly to increased cardiovascular risk in patients with HCV MC [49, 50].

#### HBV‐related MC (HBV MC)

The role of HBV as an etiologic agent for MC was first proposed over 40 years ago [51]. Subsequent studies report that the prevalence of chronic HBV infection in Europe (mainly Italy and France) ranges from 0.5% to 5.5% [36, 52, 53, 54], whereas HBV‐related MC (HBV MC) appears more common in China [55, 56]. Similar to HCV, HBV can infect both liver and lymphoid cells. Approximately 20% of patients with HBV infection develop extrahepatic manifestations, including MC vasculitis, polyarteritis nodosa, glomerulonephritis, and NHL [57]. Although the number of HBV‐associated cases is smaller, they are clinically significant.

**Table 4:** The most common clinical manifestations observed in mixed cryoglobulinemia.

Most frequent features of Types II and III (mixed; MC)
Cutaneous vasculitis (i.e., purpura)
Arthralgias and arthritis in the proximal interphalangeal and metacarpophalangeal joints (fingers, hands, and toe joints), knees and ankles
Weakness
Renal disease
Peripheral neuropathy (numbness hands and feet)
Myalgias

MC serological stigmata
Mixed CGs
RF+
Low C4
Autoantibodies (ANA, ENA, AMA, ASMA, anti‐GOR)

Most studies indicate that 40%–100% of cases present with the classic triad described by Metzler and Franklin: palpable purpura on the legs, asthenia, and arthralgia [53, 56, 58, 59]. The purpura is typically orthostatic and bilateral, and the arthralgias are symmetric, non‐deforming, and mainly involve the knees and hands. Skin ulcers occur in 14%–30% of cases. Renal involvement is observed in approximately 10% of patients, most commonly presenting as MPGN. Peripheral nervous system involvement, ranging from 22% to 61%, often manifests as distal sensory polyneuropathy that can progress to motor neuropathy. Sicca syndrome and Raynaud's phenomenon are reported in about 20% of patients. Additionally, indolent low‐grade B‐cell NHL is observed in about 10% of patients [10].

#### Mechanisms of MC vasculitis

Regarding the potential mechanisms underlying MC vasculitis, available data derive almost exclusively from studies on the HCV‐related form, which is therefore considered a key model for understanding the pathogenetic processes involved in this condition.

Over more than three decades of research, numerous and diverse pathogenetic hypotheses and mechanisms have been proposed. The fundamental pathogenic pathway is based on the unique characteristics of the virus—both structural and functional—including its ability to infect lymphoid cells, as discovered early after the virus's identification [60]. These properties enable HCV to induce a spectrum of B‐cell LPDs, ranging from isolated polyclonal hypergammaglobulinemia with detectable cryoglobulins in serum to CV and, ultimately, B‐cell malignancies [19, 30, 46, 61, 62].

**Fig. 2:**
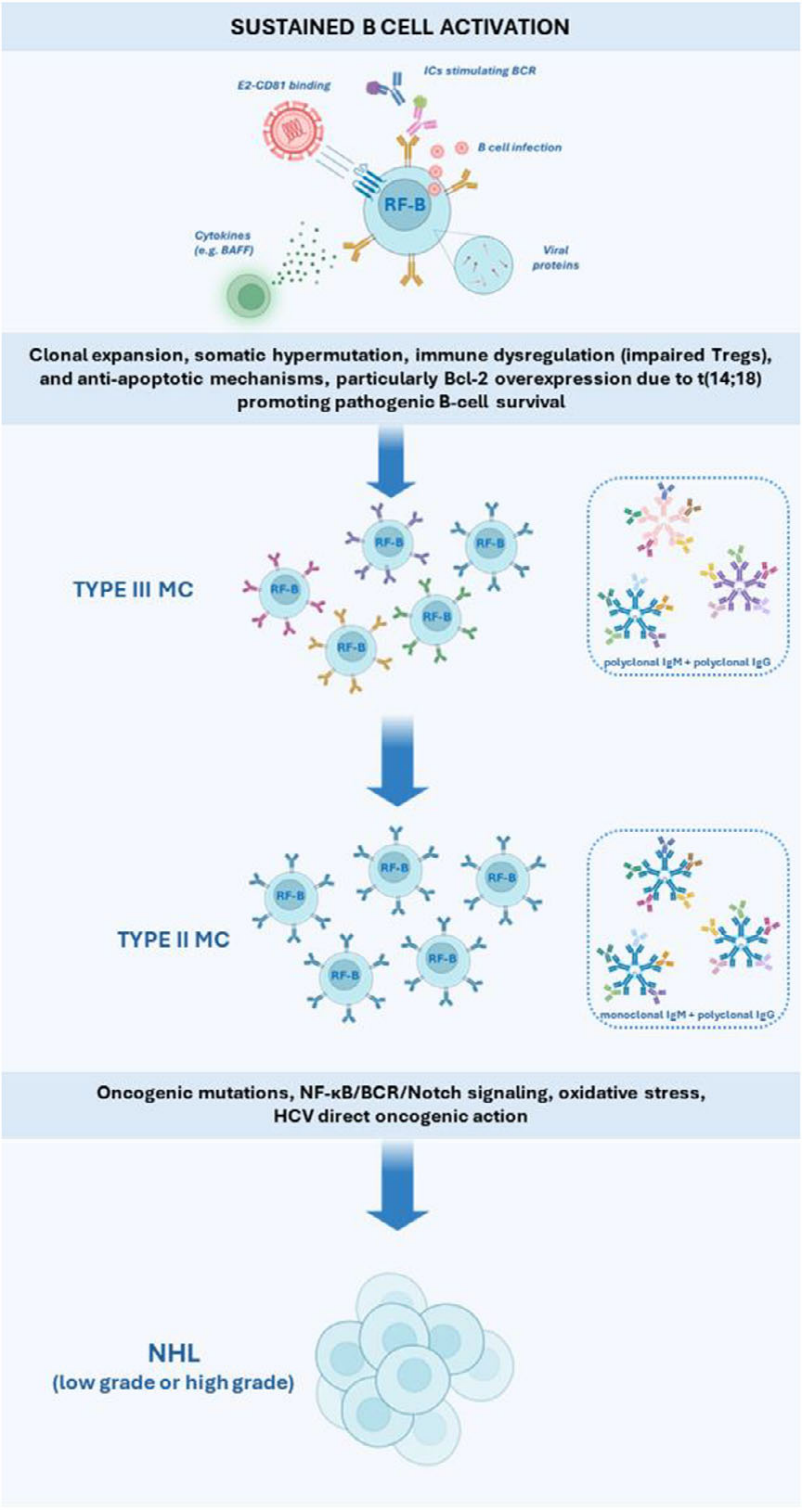
Hepatitis C virus (HCV)‐related mixed cryoglobulinemia (MC) represents the main model for understanding pathogenesis. Persistent B‐cell stimulation (driven by E2–CD81 interaction, viral infection of B cells, viral proteins, and cytokines—notably B‐cell activating factor [BAFF]) promotes clonal expansion, somatic hypermutation, and immune dysregulation (impaired Tregs). Anti‐apoptotic mechanisms, particularly Bcl‐2 overexpression due to t(14;18) translocation, further sustain pathogenic B‐cell survival. Over time, immune complexes evolve from polyclonal (Type III) to monoclonal (Type II), with expansion of IgM RF+ clones. “No‐return” events (oncogenic mutations, NF‐κB/BCR/Notch signaling, oxidative stress) may lead to lymphoma. Pathogenic clones with atypical memory phenotype (CD21^−^CD27^+^IgM^+^T‐bet^+^) also occur in non‐HCV‐related (essential and autoimmune‐associated) MC. Even after HCV eradication, RF+ clones may persist, with possible reactivation of vasculitis. RF‐B, RF‐producing B cells; NHL, non‐Hodgkin lymphoma.

Observations of the association between HCV MC and various factors spanning multiple fields—such as genetics, epigenetics, and immunology (e.g., cytokines and cell phenotypes)—suggest their key involvement in disease pathogenesis (Fig. 2). For some factors, it is difficult to distinguish between the alterations involved in the initial induction of the disease and those that arise secondarily as a consequence of its establishment.

A potential interpretation of the pathogenetic process leading from HCV infection to the development of MC and other B‐LPDs (see Fig. 2) is based on the persistent and potent stimulation of the B‐cell compartment. In the context of HCV infection, this stimulation may result from a combination of factors, including the virus's ability to infect B cells; the E2‐CD81 binding (that lowers the activation threshold, reduces the B‐cell threshold for activation and proliferation, and induces immunoglobulin somatic hypermutation, resulting in hypergammaglobulinemia), a direct action of HCV proteins, and the effect of several cytokines, first of all the B‐cell activating factor (BAFF) [63, 64, 65]. The persistence and accumulation of B‐cell clones are further promoted by anti‐apoptotic mechanisms, such as Bcl‐2 overexpression due to  *t*(14;18translocation (that may be favored by intense B‐cell activation and proliferation), and by immune dysregulation, including impaired regulatory T‐cell function. Over time, cryoglobulins evolve from polyclonal (Type III) to monoclonal (Type II), reflecting the expansion of IgM RF‐producing B‐cell clones. Additional “no‐return” events (oncogenic mutations, dysregulated signaling pathways—NF‐κB, BCR, NOTCH—and oxidative stress) may ultimately lead to lymphoma, sometimes independently of CG [66, 67]. Recently, C. Young et al. proposed a three‐step process in which somatic mutagenesis converges on a single B cell to generate pathogenic clones able to produce high‐affinity insoluble cryoglobulins, without evidence of molecular mimicry against the HCV E2 antigen as the initiating trigger [68]. The possibility of direct oncogenic action of the virus on infected cells, as demonstrated in laboratory experiments, has also been proposed according to the so‐called hit‐and‐run theory [66]. It is noteworthy that significant modifications in the B‐cell compartment can be observed not only in HCV MC but also in other variants such as the so‐called essential MC or MC associated with autoimmune conditions, more often the SS [62]. In all these conditions, B cells with an atypical memory phenotype (CD21^−^CD27^+^IgM^+^T‐bet^+^) are expanded [69, 70]. These B cells are characterized by an array of homing and inhibitory surface receptors and by impaired BCR signaling and poor proliferative responses to the triggering of BCR and of TLR9, although it has been shown that TLR9 promotes enhanced secretion of RF‐type IgMs in patients with HCV MC [71, 72]. In HCV‐cured patients, despite the partial reversion of the anergic signature of CD21^low^ B cells, RF‐expressing B‐cell clones persist over time [73]. It has been demonstrated in vitro that autoantigens, in the form of immune complexes and CpG of microbial origin, may cooperate to support the survival of pathogenic B cells in the absence of HCV [73]. The role of infections as triggers of RF^+^ B‐cell clone reactivation and vasculitis relapse, despite HCV eradication, is supported by the fact that vasculitis relapse has been observed after SARS‐CoV‐2 and other systemic infections [74].

It is important to emphasize that the aforementioned mechanisms may be, either wholly or in part, involved in HCV‐related lymphomagenesis, even independently of the development of MC. According to some lines of thought, the choice of the evolutionary pathway largely depends on the presence of a genetically predisposed background [30, 75, 76].

## Therapeutic approaches

### Type I CG

Patients with symptomatic Type 1 CG can pose difficult therapeutic dilemmas. Due to the rarity of this condition and its association with various hematological disorders, no prospective controlled studies are available. Only a few retrospective observational studies, with limited sample sizes, have been published [17, 23, 24, 77, 78, 79, 80, 81]. Consequently, no standard of care or international guidelines have been established for the treatment of Type I CG, and recommendations are derived primarily from expert opinion [17]. The management of Type I CG focuses on controlling the severity of vasculopathy and treating the underlying hematologic malignancy. Given its potential life‐threatening nature, early and aggressive intervention is generally required [14, 17, 23]. Treatment strategies may involve the use of glucocorticoids (GCs), plasma exchange, and immunosuppressive agents. In patients requiring systemic therapy (e.g., due to the presence of skin ulcers), targeting the B‐cell clone (plasma cell or a lymphoplasmacytic cell) is based on the consideration that such clonal expansion may lead to more severe disease manifestations. Nevertheless, evidence suggests that although treating the underlying LPD generally leads to symptom improvement, it results in a complete resolution of CG in only about half of cases [80]. When there is no definitive indication for hematologic intervention, evaluating the isotype of the monoclonal immunoglobulin becomes essential. In fact, in cases involving the IgM isotype, the associated lymphoplasmacytic cells typically express the CD20 surface antigen, making them potential candidates for therapy with rituximab (RTX) or other anti‐CD20 monoclonal antibodies, either as monotherapy or combined with agents such as bendamustine or cyclophosphamide. Additionally, given that most IgM immunoglobulins are present in circulation, plasmapheresis may offer therapeutic benefit as well [14]. By contrast, in cases of Type I CG associated with non‐IgM isotypes (e.g., IgG or IgA), therapeutic regimens incorporating agents that target plasma cells (i.e., bortezomib or anti‐CD38 monoclonal antibodies such as daratumumab or isatuximab) may prove effective. Plasmapheresis is frequently employed as part of the treatment strategy, along with the use of GCs and various immunosuppressive drugs in combination [6, 23]. In patients presenting with mild and isolated symptoms, supportive non‐pharmacologic strategies may be adequate. These may involve advising the avoidance of cold environments, promoting sufficient rest, and suggesting the use of compression stockings for managing mild‐to‐moderate gravity‐dependent purpura [23].

### Type II and Type III MC

Most of the available data concerning the therapeutic approach to Type II or III MC derives from the treatment of MC associated with hepatitis viruses, particularly following the introduction of more modern antiviral therapy (AVT), such as direct‐acting antivirals (DAAs) for HCV infection and nucleos(t)ide analogs (NAs) for HBV infection.

Overall, therapeutic regimens involve eliminating viral triggers in cases with infectious causes, along with tailoring treatment strategies based on the severity of the vasculitis [30, 82, 83]. These latter—which were the only therapeutic options available prior to the discovery of the link with the infection—may include various combinations of GCs, immunosuppressive agents, and plasma exchange. Their use, in the absence of AVT, has generally led to inconsistent outcomes and a poor prognosis [14, 84].

#### Treatment of HCV‐related MC (MC‐HCV)

The discovery of the association between HCV and MC—along with studies conducted following the introduction of AVT—has demonstrated significant clinical improvement resulting from viral eradication, which, in the context of HCV infection, corresponds to the sustained virological response (SVR).

First‐generation interferon (IFN)‐based regimens were limited by IFN neurotoxicity, myelosuppression, and ribavirin‐induced hemolysis [32, 39, 85]. Clinical remission correlated with sustained virologic response (SVR), although some discordant data exist [32]. In a prospective study of more than 400 HCV patients with an 8‐year post‐AVT follow‐up (F‐U), CV symptoms resolved in 57% of SVR cases, but MC was a negative prognostic factor for virological response [86, 87]. In view of the overall complexity of treatment‐related issues, IFN‐based AVT was recommended only for mild‐to‐moderate CV, excluding severe forms [44].

The introduction of DAAs radically changed the scenario, greatly enhancing both the efficacy and safety of etiological treatment [61, 88]. Indeed, with the introduction of IFN‐free AVT, SVR rates reached levels of around 90% of patients, along with a significant reduction in side effects. Moreover, no significant difference in SVR rates was observed in patients with MC compared to HCV patients without MC [89, 90, 91]. Furthermore, high clinical and immunological effectiveness was confirmed in patients with HCV‐related CV (HCV‐CV) [91, 92] (Table 5). Nevertheless, some patients with HCV‐CV do not achieve a complete clinical and immunological response, or, in some cases, a recurrence of vasculitis during F‐U is observed (see also below) [85, 86, 87, 91, 93].

**Table 5:** Hepatitis C virus (HCV) cryoglobulinemic vasculitis response to direct‐acting antivirals (DAAs) and suggested adverse predictors.

Reference	Pts no.	Overall clinical response (%)	Overall immunological Response	F‐U mean (range)	Suggested adverse predictors of poor response	
					Clinical	Laboratory
*Gragnani and Visentini [89]	44	100	73.5%	24 weeks	Salivary glands and peripheral nerve damage	MBL
Lauletta [114]	22	86.4	77.3%	12 weeks	Peripheral neuropathy Glomerulonephritis Lymphoma	ND
Emery [115]	11 DAA 7 peg‐IFN	62	80%	20 weeks (14–86)	ND	ND
Mazzaro [116]	22	75	68%	48 weeks	Peripheral nerve damage	ND
*Gragnani [88]	85	96.7	87.5%	65 weeks (36–92)	Severe, long‐lasting CV Kidney involvement	ND
Passerini [117]	35	65.7	68.5%	32 weeks	Neurologic symptoms Kidney involvement Lymphoma	ND
Cacoub [118]	148	95.2	53.1%	61 weeks	CV severity (*p* = 0.03) Neuropathy (*p* = 0.02)	ND
Pozzato [93]	67	60	60%	96 weeks	Salivary gland and peripheral nerve damage	MBL (2)
Gragnani [61]	98	53	ND	48 weeks (24–96)	ND	MBL (*p* = 0.04) altered κ/λ ratio (*p* = 0.003) *t*(14;18)+ (*p* = 0.02) RF + (*p* = 0.02) NOTCH4 polymorphism (*p* = 0.01)
*Gragnani [105]	108	50	ND	137 weeks (72–260)	Weakness (0.03) Sicca syndrome (*p* = 0.0001) Neuropathy *p* = 0.0029) CV severity index (*p* = 0.02) Global Severity Index (*p* = 0.005)	ND
*Kondili [91]	523	88	ND	15 months	Age (*p* = 0.002)* Renal involvement	ND

*Note*: Overall clinical response: the percentage of patients experiencing improvement of most CV symptoms, variably described by different authors; for example, in * the overall clinical response includes the complete clinical response (improvement of all pre‐AVT vasculitis symptoms/signs), the full complete response (complete disappearance of all pre‐AVT vasculitis symptoms/signs) and the partial clinical response (improvement of more than half of pre‐AVT vasculitis symptoms/signs). Overal immunological response: the percentage of patients experiencing both complete and partial immunological resonse

Abbreviations: CV, cryoglobulinemic vasculitis; F‐U, follow‐up; IFN, interferon; MBL, monoclonal B‐cell lymphocytosis; RF, rheumatoid factor.

The clinical response (the response of symptoms characterizing the CV, such as purpura, weakness, arthralgia, and neuropathy) was generally described as complete (improvement of all symptoms: CR), partial (the improvement of at least half of the symptoms: PR), and null response (NR), including all the rest. In some studies, the complete disappearance of all symptoms and laboratory data was defined as “full complete response” (FCR: restitutio ad integrum) [89, 91]. The immunological response (the effect of therapy on the cryoglobulin, serum RF, and C4 component of complement levels) was also generally described as complete (CR), partial (PR), and null response (NR), with variable criteria in different studies.

The overall clinical response (including both CR/FCR and PR) was generally observed in most CV after SVR, reaching even 100% of patients in initial studies characterized by limited populations and F‐U times (Table 5).

Interestingly, unlike IFN‐based AVT—where symptoms and quality of life usually decreased during treatment—an improvement was usually noticed early during DAA‐based therapy [30, 90]. Purpura is one of the symptoms that responds better and more rapidly to viral eradication, persisting less than other symptoms such as arthralgia, fatigue, nephropathy, neuropathy, and sicca syndrome. These clinical findings, along with laboratory evidence, have collectively underscored the necessity of AVT in order to eliminate the infection in affected patients [92]. Laboratory evidence includes the regression of classical laboratory markers of MC, such as cryocrit, RF, and complement consumption. Cryocrit levels decreased in all cases, with a complete disappearance of cryoglobulins in the serum in only about 30% of patients; RF levels declined in some patients, returning to normal in approximately 25% of cases; and elevated C4 complement levels have been reported in most studies, although they remained persistently below normal throughout treatment in several ones [10, 58, 59]. The potential regression of B‐cell clonal expansion has also been observed, leading to the disappearance of detectable *t*(14;18) translocation in circulating B cells [94, 95] and the restoration of immune balance between elevated atypical memory B cells, pathogenic T lymphocytes, and reduced regulatory T cells [96]. Uncontrolled B‐cell proliferation may progress to overt lymphoma that warrants specific clone‐targeted treatment [31] (Table 5 and Fig. 1).

In one study, patients with HCV‐associated CV and lymphoma obtained SVR and good CV response after AVT with DAAs but unsatisfactory hematological response with persistence of molecular B‐cell clonality [97].

**Fig. 3:**
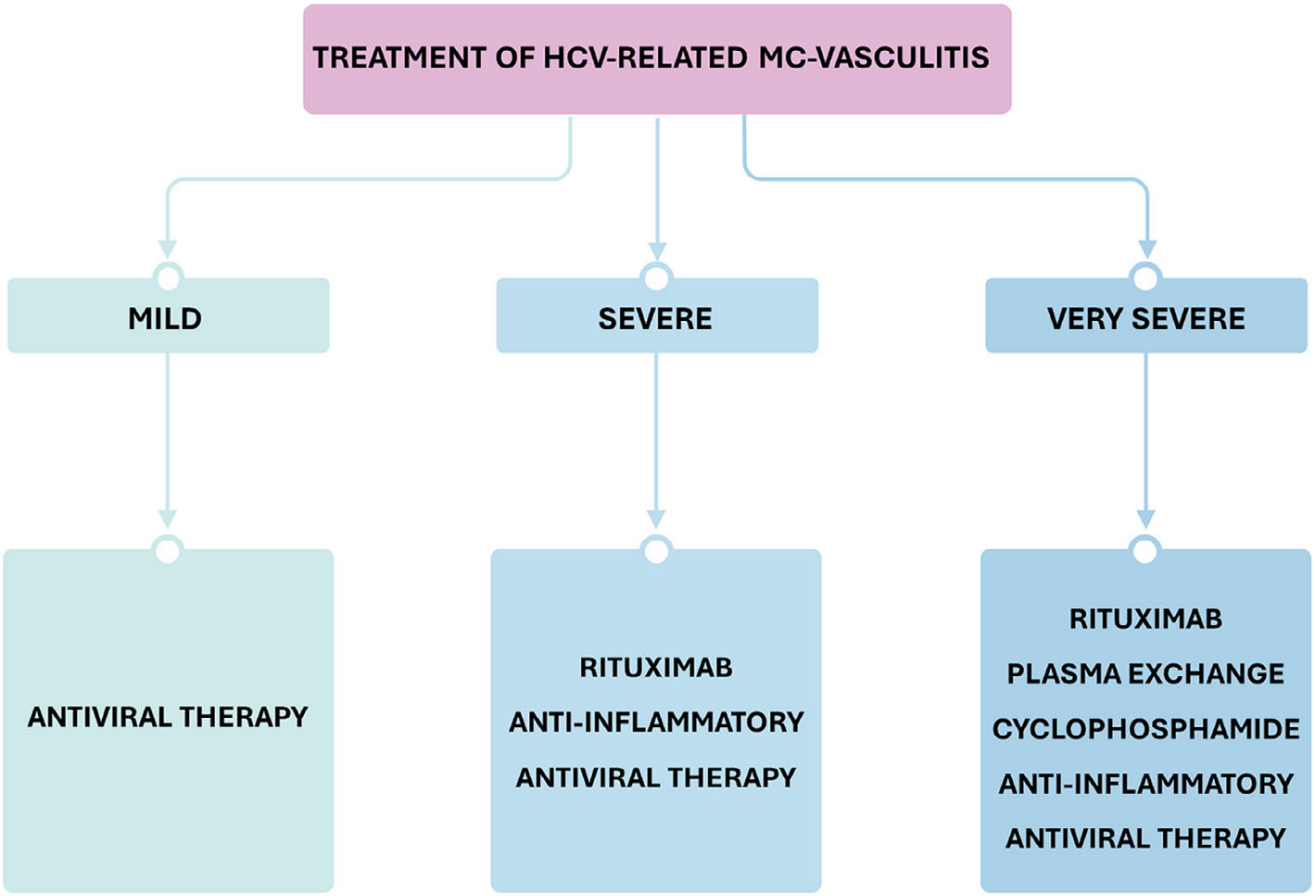
The currently available evidence allows for an outline of a simplified therapeutic approach to cryoglobulinemic vasculitis (CV). Mild forms (e.g., non‐necrotic purpura, arthralgia, myalgia, or purely sensory neuropathy) often resolve with direct‐acting antivirals (DAAs) alone. Moderate–severe forms (e.g., glomerulonephritis with impaired renal function, skin necrosis, peripheral neuropathy with motor involvement) may require adjunctive non‐etiologic therapies tailored to the patient, mainly rituximab /anti‐CD20 mAbs, and anti‐inflammatory agents such as glucocorticoids (most frequently used) and colchicine. Very severe/life‐threatening cases (e.g., rapidly progressive glomerulonephritis, central nervous system, digestive, cardiac, or pulmonary involvement) may also need immunosuppressants (e.g., cyclophosphamide) and rapid interventions such as apheresis. In addition to these main interventions, analgesics (NSAIDs, acetaminophen, gabapentin, pregabalin, opioids, amitriptyline, and duloxetine) and supportive measures such as a low‐antigen diet may also be helpful. In particular, neuropathic pain may benefit from gabapentin or pregabalin, and in some cases, amitriptyline or duloxetine, which can also contribute to reducing glucocorticoid exposure.

Taken together, the experience gained so far indicates that AVT with DAAs is highly effective in the majority of patients with mild‐to‐moderate symptoms (e.g., arthralgia, myalgia, non‐necrotic purpura, or purely sensory neuropathy). However, AVT alone may be insufficient in cases of more severe vasculitic manifestations. In these patients, additional measures beyond viral clearance are often required, with dosages and regimens tailored to the individual. Such clinical scenarios include complications such as glomerulonephritis with impaired renal function, skin necrosis, or peripheral neuropathy with motor involvement [90] (Fig. 3). Among these adjunctive options, biological agents such as RTX or other anti‐CD20 monoclonal antibodies are generally considered the second‐line treatment of choice after viral eradication, whereas anti‐inflammatory agents such as GCs (most frequently used) and colchicine are also employed. These interventions are usually sufficient; however, in very severe or life‐threatening cases (e.g., rapidly progressive glomerulonephritis or involvement of the CNS, gastrointestinal tract, heart, or lungs), treatment with immunosuppressants (e.g., cyclophosphamide) and rapid‐acting measures such as plasma exchange is indicated (Fig. 3). Additional benefit in patient management may be provided by analgesics and symptomatic agents (nonsteroidal anti‐inflammatory drugs ‐NSAIDs‐., acetaminophen, gabapentin, pregabalin, opioids, and amitriptyline) as well as supportive/non‐pharmacological measures (including, for some authors, a low‐antigen diet). Notably, analgesics and NSAIDs—particularly gabapentin, pregabalin, and, in some cases, amitriptyline or duloxetine—may help relieve neuropathic pain and may also contribute to reducing GC exposure.

Among non‐etiologic therapies, RTX deserves special mention, as its efficacy has been consistently documented [98]. In a study involving patients who were not eligible for viral eradication with the available AVT, treatment with RTX was associated with improvement not only in vasculitic symptoms but also in HCV‐related cirrhosis [99]. Several different clinical trials and observational studies have been conducted to assess this approach [100, 101, 102]. Some studies have reported a higher incidence of significant adverse effects when higher single‐dose regimens were used (i.e., two 1000‐mg doses of RTX administered 2 weeks apart). Consequently, the authors recommend adherence to the standard protocol of four weekly infusions of 375 mg, along with standard premedication, including GC, paracetamol, and chlorphenamine, prior to each infusion [103]. For very severe manifestations, plasma exchange and high‐dose GC pulses may be added [83].

##### Predictive factors of response to AVT in HCV‐related CV

Recent long‐term F‐U studies of patients with HCV‐CV treated with DAAs have confirmed a high rate of persisting clinical response. However, they also highlight that CV manifestations can persist, relapse, or even appear de novo posttreatment. These events often resemble autoimmune or autoinflammatory conditions, including episodes of autoimmune hepatitis [85, 92, 104].

**Fig. 4:**
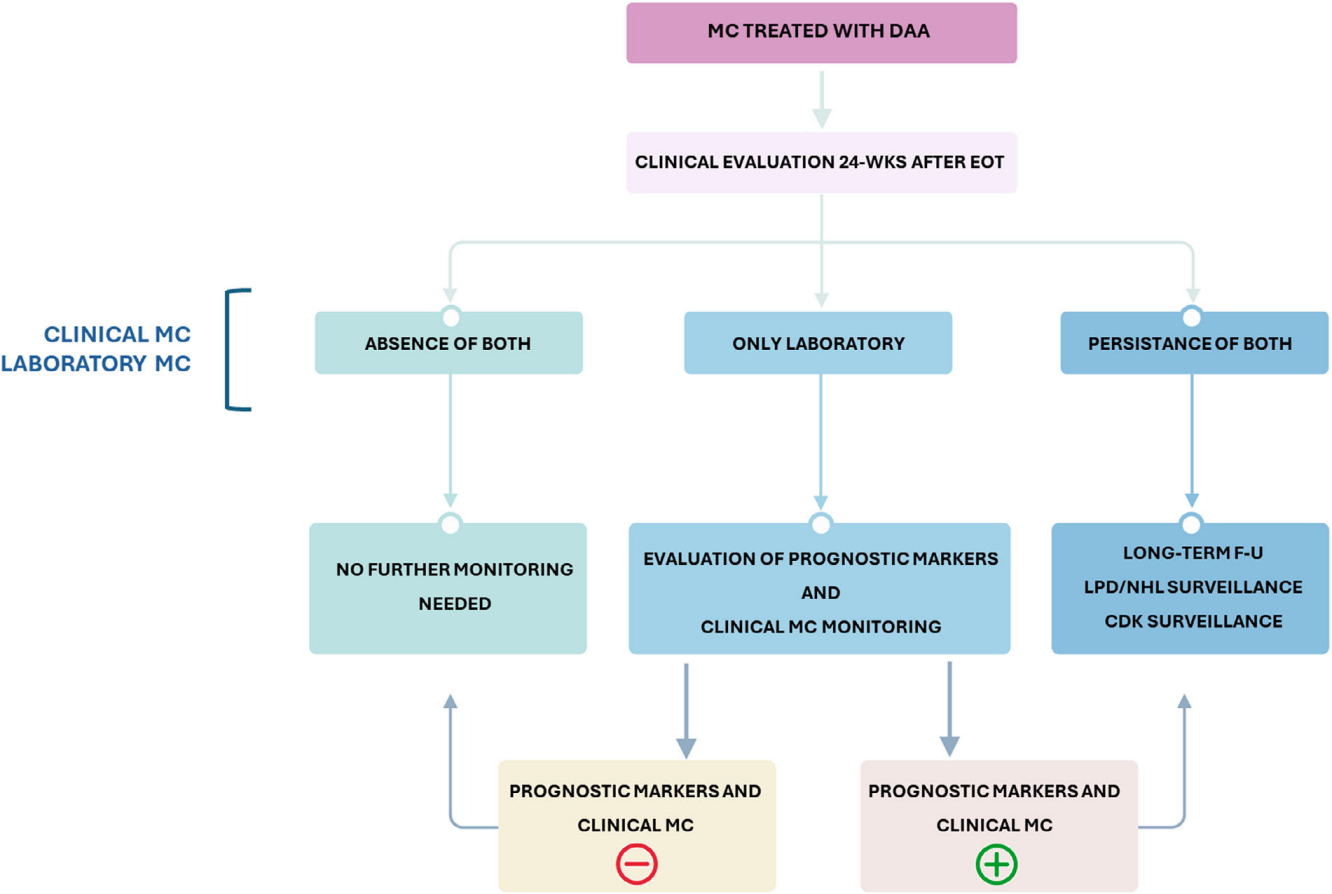
After sustained virological response (SVR), cryoglobulinemic vasculitis (CV) patients may show (i) complete clinical and laboratory remission, (ii) persistence of laboratory markers alone (cryoglobulins with or without high rheumatoid factor (RF) values, complement consumption), or (iii) persistence of both clinical and laboratory abnormalities. The follow‐up (F‐U) should therefore be personalized in timing and intensity, considering relapse risk and prognostic factors. Clinical + laboratory persistence requires long‐term monitoring, with particular attention to LPDs and renal disease. Laboratory persistence: It is advisable to monitor over time for the possible emergence of clinical manifestations and negative prognostic factors. If one or more of these criteria become positive, continued long‐term F‐U is warranted. Dedicated studies are currently ongoing to evaluate whether persisting cryoglobulinemia in the absence of symptoms should warrant long‐term monitoring. In the absence of established data, it has been recommended that, in the absence of both clinical manifestations and negative prognostic factors, discontinuation of regular patient monitoring may be considered [92]. No clinical or laboratory activity: In the absence of prognostic risk factors, discontinuation of regular monitoring may be considered. Still available prognostic factors (see Table 5 and Refs. [89, 108]) include laboratory, demographical, clinical markers, and specific scores. Finally, an accurate F‐U should also consider the occurrence of possible triggers of mixed cryoglobulinemia (MC) flares, such as major infectious episodes, that is, pneumonia and COVID‐19 [85], and some vaccinations (see Table 5). Notably, vaccine‐related flares are rarer and milder than those following natural infections, reinforcing vaccination as a safe and recommended strategy.

Several studies have shown potential correlations between cryoglobulinemic flares and conditions characterized by intense B‐cell stimulation, including lung cancer, severe pneumonia, influenza, and vaccinations such as those against influenza and SARS‐CoV‐2 [74, 85, 86]. Importantly, CV flares after SVR do not necessarily indicate a severe clinical course as seen in idiopathic cases. Most flares tend to be transient and respond well to treatment with GCs, immunosuppressants, or RTX [85]. In addition, it has been shown that the risk of CV flare following SARS‐CoV‐2 vaccination is consistently lower than that observed after natural infection. Thus, the concept remains that vaccination in these patients is not only not contraindicated but also recommended, as in all other conditions of iatrogenic immunosuppression. Certain demographic, clinical, and laboratory features have been identified as predictors of poor response or relapse after SVR and clinical remission. These include age and vasculitis severity (i.e., high CV severity index and global severity index) [61], together with hematological and genetic determinants (monoclonal B‐cell lymphocytosis [MBL], altered serum *κ*/*λ* ratio, *t*(14;18), RF, and NOTCH4 polymorphism) [92, 105, 106, 107] (Table 5). Assessing these factors—individually or in combination—can help guide F‐U strategies (Fig. 4).

Recently, important real‐world clinical data have emerged from large multicenter studies.

In a nationwide prospective multicenter study conducted in Italy, 1078 patients with HCV‐related CG were enrolled, including 523 with clinically overt CV. CV patients achieved SVR after DAA and were followed for 13–27 months after therapy (mean F‐U 15 months). None had concomitant NHL. During F‐U, 13% experienced relapse, which was transient in approximately 70% of cases. Predictors of poorer clinical response and relapse included indicators of disease severity (particularly renal involvement and older age) (Table 4). Multivariable analysis of laboratory data showed that elevated pretreatment RF levels (likely indicative of substantial expansion of RF‐producing B‐cell clones) were an independent prognostic factor for clinical deterioration or relapse during F‐U.

Of interest are the findings of a large retrospective multicenter study by Fayed et al. [108], which included predominantly patients from Egypt (79.8%; the remaining from France and Italy). A total of 913 patients were followed, of whom 911 had achieved SVR after DAAs, 20 had concomitant low‐grade B‐cell NHL, and relapse occurred in 12.6% (115/913) of patients. Male sex and markers of advanced CV (baseline skin ulcers, renal involvement, and peripheral neuropathy at the end of AVT) were identified as independent predictors of relapse. Relapses were managed with GCs (90.9%), alone or combined with plasma exchange (50%), cyclophosphamide (37.3%), or RTX (6.4%). Relapses were generally moderate‐to‐severe and negatively impacted 24‐month survival, mainly due to infections related to immunosuppressive therapy.

##### Clinical management of HCV MC after DAA therapy

Overall, MC patients following SVR may exhibit heterogeneous patterns, ranging from the complete disappearance of both clinical and laboratory data to the persistence of only laboratory data (e.g., CG and/or FR and/or complement consumption) or the persistence of both clinical and laboratory data. A rational clinical approach should take these differences into account by assessing a personalized F‐U with regard to both the timing of the checks and the accuracy. This tailoring should consider the occurrence of clinical relapses and the presence of different prognostic indices (Fig. 4). According to this criterion, in cases where both laboratory markers and clinical symptoms persist, long‐term F‐U is recommended, with particular attention to the potential development of LPDs and/or renal impairment. In cases where only laboratory markers of CG persist after viral eradication, it is advisable to monitor over time for the possible emergence of clinical manifestations and negative prognostic factors. If one or more of these criteria become positive, prolonged long‐term F‐U is warranted. In theory, persisting CG even in the absence of symptoms should always be regarded as an abnormality indicating the persistence of a B‐cell clonal expansion and, as such, warrants long‐term monitoring. Dedicated studies are currently ongoing in this regard. However, in the absence of established data on its actual clinical significance in all cases, and considering the economic implications of prolonged accurate F‐U, it has been recommended that, in the persistent absence of both clinical manifestations and negative prognostic factors, discontinuation of regular patient monitoring may be considered [92]. Still available prognostic factors (see Table 5 and Ref. [89]) include laboratory and demographic/clinical markers and specific scores. Laboratory markers include “clonality biomarkers” [61] (free light chain K/L ratio in serum, *t*(14;18) and MBL in PBMC); “genetic biomarkers” [61] (Notch4 polymorphism); and high cryocrit and high RF values [91]. Demographic/clinical biomarkers include advanced age, presence of renal involvement, neuropathy, weakness, and sicca syndrome. Specific scores include CV and Global Severity Index (see Table 5). Finally, an accurate F‐U should also consider the occurrence of possible triggers of MC flares, such as major infectious episodes, that is, pneumonia and COVID‐19 [85]—and some vaccinations (see Table 5). Notably, CV flares following vaccination occur at significantly lower rates than those triggered by natural infections and are often transient, reinforcing the importance of vaccination.

These recommendations apply to patients who, before starting AVT, had a clinically evident CV. Conversely, there is no evidence on how to manage F‐U in patients who, prior to SVR, had no CV but exhibited only laboratory abnormalities (presence of cryoglobulins with or without high RF levels or complement consumption) without clinical symptoms. Although the persistence of cryoglobulins after SVR, even in such conditions, theoretically suggests a potential risk factor, both in terms of flares following strong B‐cell stimulation and of a possible evolution toward malignant B‐cell LPDs during long‐term F‐U [10, 93, 97], appropriate studies will be required to confirm the validity of this assumption.

#### Treatment of HBV‐related MC

In the case of HBV‐related MC as well, growing evidence supports the beneficial effects of AVT, despite some notable differences compared to HCV‐related cases. Several studies have shown that AVT with NAs is able to effectively suppress HBV replication and can provide a good clinical response in patients with mild‐to‐moderate symptoms, such as purpura, asthenia, and arthralgia [10, 38, 53, 56, 58, 59]. AVT with IFN has generally been less well tolerated and less effective, and in some cases has even led to significant worsening of symptoms [38]. In some cases, NAs have been combined with GCs to enhance control of vasculitic symptoms. However, the use of RTX has been associated with a significant risk of HBV reactivation and its potential complications; therefore, it should be used with caution. Nonetheless, RTX can be highly valuable in select cases and is now permitted when co‐administered with AVT based on new‐generation NAs (e.g., entecavir, tenofovir disoproxil fumarate, and tenofovir alafenamide), which enables effective viral control. This approach is typically employed in severe cases, such as in patients with skin ulcers, motor‐predominant peripheral neuropathy, or nephropathy, or in patients who are non‐responders or experience relapse. In such scenarios, RTX—either alone or in combination with plasma exchange—may be safely administered alongside NAs.

This comprehensive therapeutic strategy allows for effective suppression of HBV replication while concurrently addressing the systemic manifestations of HBV‐related CV.

##### Effect of antiviral therapy on symptoms of HBV‐related MC

During treatment with NAs, approximately 80% of patients experienced improvement or resolution of purpura, asthenia, and arthralgia within 12 months [10, 53, 58]. In some cases, NAs were combined with GCs to enhance control of vasculitic symptoms. Specifically, the response of leg ulcers to NAs alone was limited. Only a small number of patients achieved complete ulcer resolution with NA monotherapy, whereas others required plasma exchange followed by RTX to achieve clinical improvement [10]. Peripheral neuropathy improved in approximately 40% of patients treated with NAs alone. However, those with severe sensorimotor neuropathy did not respond adequately and required RTX therapy [10]. Renal involvement showed no significant improvement with NAs alone. Patients with renal manifestations typically required plasma exchange followed by RTX infusions [10, 53, 56]. In some cases, renal failure progressed despite immunosuppressive therapy, ultimately requiring dialysis [56]. In a study of non–HCV MC, including HBV‐associated MC, male patients were found to have the poorest outcomes [14]. Only very limited data are available regarding the response to AVT in HBV‐associated lymphoma. One low‐grade NHL case treated with NAs achieved HBV–DNA suppression and purpura improvement but did not show a hematologic response. The patient later received RTX, achieving a partial hematologic response [10].

##### Duration of antiviral therapy and relapse prevention

Unlike AVT for HCV infection—which is time‐limited and leads to viral eradication in the vast majority of cases—the currently available therapies for HBV, although effective in suppressing viral replication, do not achieve viral eradication. As a result, they often require prolonged, sometimes lifelong, treatment, even after vasculitis symptoms have been resolved. The criteria for safely discontinuing HBV therapy without subsequent viral reactivation remain a matter of ongoing debate. A case of HBV MC relapse has been reported following discontinuation of entecavir, which led to reactivation of viral replication, recurrence of vasculitis symptoms, and elevated cryocrit levels. Retreatment with entecavir successfully restored clinical remission, coinciding with suppression of viral replication to undetectable levels [109]. Based on current knowledge, discontinuation of NA therapy can be considered only in patients who have achieved complete remission of vasculitis, loss of HBsAg, and HBsAg seroconversion. Interestingly, a case was reported in which a patient with HBV MC—despite maintaining undetectable HBV–DNA levels under NA therapy—experienced a vasculitis flare following influenza vaccination. The symptoms were effectively managed with GCs, and HBV DNA remained undetectable throughout the course of treatment [110].

##### Effects of AVT with NAs on immunological parameters

Similar to what has been described in HCV MC treatment with NAs in HBV‐related cases was associated with a reduction in cryocrit levels in all patients, with approximately 30% achieving complete clearance of serum cryoglobulins. RF levels decreased in some patients, returning to normal in about 25% of cases. However, serum C4 levels remained persistently low throughout the course of treatment [10, 58, 59].

#### Treatment of noninfectious MC

In cases of noninfectious MC, treatment is guided by the underlying pathophysiological mechanism, which typically involves an autoimmune disorder characterized by excessive B‐cell activity (such as SLE or SS) [111, 112]. GCs, with or without methotrexate, are generally effective for cutaneous–articular forms of noninfectious MC, whereas colchicine may be used for isolated cutaneous vasculitis [113]. In severe cases, particularly those with renal or neurologic involvement or refractory cutaneous‐articular manifestations, RTX is the treatment of choice. Cyclophosphamide may be considered in refractory cases, and plasma exchange is reserved for severe or life‐threatening complications [14].

## Conclusions and future perspectives

In the case of Type I CG, the wide spectrum of underlying hematologic disorders presents substantial challenges for research. Concerning the MC, studies conducted over the past decades have made significant progress following the discovery of traceable and targetable viral agents, opening the door to innovative therapeutic strategies. With the global decline in HCV prevalence, the overall incidence of MC has decreased, resulting in a relative increase in the proportion of noninfectious forms.

Past and future developments are expected to substantially improve the management of both virus‐related forms of MC, especially those persisting after AVT, and non‐virus‐related forms.

Future efforts should prioritize translational research and multicenter, randomized controlled trials focusing on noninfectious MC and Type I CG, with the ultimate goal of improving patient outcomes and long‐term prognosis.

## Conflict of interest statement

Luca Arcaini: EUSA Pharma, Novartis, Kite, Beigene, Abbvie (Speaker Bureau); Roche, Janssen‐Cilag, Verastem, Incyte, EUSA Pharma, Celgene/Bristol Myers Squibb, Kite/Gilead, ADC Therapeutics, Novartis (Participation on a Data Safety Monitoring Board or Advisory Board); Roche, Astra Zeneca (Support for attending meetings and/or travel); the authors declare no conflicts of interest.

## References

[1] Brouet JC , Clauvel JP , Danon F , Klein M , Seligmann M . Biologic and clinical significance of cryoglobulins. A report of 86 cases. Am J Med. 1974;57(5):775–88. 4216269 10.1016/0002-9343(74)90852-3

[2] Fadda P , La Civita L , Zignego AL , Ferri C . [Hepatitis C virus infection and arthritis. A clinico‐serological investigation of arthritis in patients with or without cryoglobulinemic syndrome]. Reumatismo. 2002;54(4):316–23. 12563365 10.4081/reumatismo.2002.316

[3] Roccatello D , Saadoun D , Ramos‐Casals M , Tzioufas AG , Fervenza FC , Cacoub P , et al. Cryoglobulinaemia. Nat Rev Dis Primers. 2018;4(1):11. 30072738 10.1038/s41572-018-0009-4

[4] Boleto G , Ghillani‐Dalbin P , Musset L , Biard L , Mulier G , Cacoub P , et al. Cryoglobulinemia after the era of chronic hepatitis C infection. Semin Arthritis Rheum. 2020;50(4):695–700. 32521323 10.1016/j.semarthrit.2020.05.004

[5] Ferri C , Ramos‐Casals M , Zignego AL , Arcaini L , Roccatello D , Antonelli A , et al. International diagnostic guidelines for patients with HCV‐related extrahepatic manifestations. A multidisciplinary expert statement. Autoimmun Rev. 2016;15(12):1145–60. 27640316 10.1016/j.autrev.2016.09.006

[6] Khwaja J , Vos JMI , Pluimers TE , Japzon N , Patel A , Salter S , et al. Clinical and clonal characteristics of monoclonal immunoglobulin M‐associated type I cryoglobulinaemia. Br J Haematol. 2024;204(1):177–85. 37726004 10.1111/bjh.19112

[7] Khwaja J , D'Sa S , Minnema MC , Kersten MJ , Wechalekar A , Vos JM . IgM monoclonal gammopathies of clinical significance: diagnosis and management. Haematologica. 2022;107(9):2037–50. 35770530 10.3324/haematol.2022.280953 PMC9425303

[8] Minafò YA , Monaco N , Fiorilli V , La Gualana F , D'Ambrosi M , De Renzis C , et al. Concurrence of mixed cryoglobulinaemia and cold agglutinin disease, and the putative role for a stereotyped immunoglobulin light chain. Br J Haematol. 2025;207(3):1155–8. 40566899 10.1111/bjh.20220

[9] Ferri C , Greco F , Longombardo G , Palla P , Moretti A , Marzo E , et al. Association between hepatitis C virus and mixed cryoglobulinemia [see comment]. Clin Exp Rheumatol. 1991;9(6):621–4. 1662567

[10] Mazzaro C , Maso LD , Gragnani L , Visentini M , Saccardo F , Filippini D , et al. Hepatitis B virus‐related cryoglobulinemic vasculitis: review of the literature and long‐term follow‐up analysis of 18 patients treated with nucleos(t)ide analogues from the Italian study group of cryoglobulinemia (GISC). Viruses. 2021;13(6):1032. 34070832 10.3390/v13061032 PMC8226459

[11] Desbois AC , Cacoub P , SD Cryoglobulinemia . An update in 2019. Joint Bone Spine. 2019;86(6):707–13. 30731128 10.1016/j.jbspin.2019.01.016

[12] De Vita S , Soldano F , Isola M , Monti G , Gabrielli A , Tzioufas A , et al. Preliminary classification criteria for the cryoglobulinaemic vasculitis. Ann Rheum Dis. 2011;70(7):1183–90. 21571735 10.1136/ard.2011.150755 PMC3103668

[13] Jennette JC , Falk RJ , Bacon PA , Basu N , Cid MC , Ferrario F , et al. 2012 revised international chapel hill consensus conference nomenclature of vasculitides. Arthritis Rheum. 2013;65(1):1–11. 23045170 10.1002/art.37715

[14] Cacoub P , Vieira M , Saadoun D . Cryoglobulinemia‐one name for two diseases. N Engl J Med. 2024;391(15):1426–39. 39413378 10.1056/NEJMra2400092

[15] Podell DN , Packman CH , Maniloff J , Abraham GN . Characterization of monoclonal IgG cryoglobulins: fine‐structural and morphological analysis. Blood. 1987;69(2):677–81. 3801676

[16] Strait RT , Posgai MT , Mahler A , Barasa N , Jacob CO , Köhl J , et al. IgG1 protects against renal disease in a mouse model of cryoglobulinaemia. Nature. 2015;517(7535):501–4. 25363774 10.1038/nature13868 PMC4342786

[17] Muchtar E , Magen H , Gertz MA . How I treat cryoglobulinemia. Blood. 2017;129(3):289–98. 27799164 10.1182/blood-2016-09-719773

[18] Monti G , Pioltelli P , Saccardo F , Campanini M , Candela M , Cavallero G , et al. Incidence and characteristics of non‐Hodgkin lymphomas in a multicenter case file of patients with hepatitis C virus‐related symptomatic mixed cryoglobulinemias. Arch Intern Med. 2005;165(1):101–5. 15642884 10.1001/archinte.165.1.101

[19] Cacoub P , Comarmond C , Vieira M , Régnier P , Saadoun D . HCV‐related lymphoproliferative disorders in the direct‐acting antiviral era: from mixed cryoglobulinaemia to B‐cell lymphoma. J Hepatol. 2022;76(1):174–85. 34600000 10.1016/j.jhep.2021.09.023

[20] Leung N , Bridoux F , Batuman V , Chaidos A , Cockwell P , D'Agati VD , et al. The evaluation of monoclonal gammopathy of renal significance: a consensus report of the international kidney and monoclonal gammopathy research group. Nat Rev Nephrol. 2019;15(1):45–59. 30510265 10.1038/s41581-018-0077-4 PMC7136169

[21] Zhang LL , Cao XX , Shen KN , Han HX , Zhang CL , Qiu Y , et al. Clinical characteristics and treatment outcome of type I cryoglobulinemia in Chinese patients: a single‐center study of 45 patients. Ann Hematol. 2020;99(8):1735–40. 32535708 10.1007/s00277-020-04123-1

[22] Ojemakinde K , Turbat‐Herrera EA , Zeng X , Gu X , Herrera GA . The many faces of cryoglobulinemic nephropathy: a clinico‐pathologic study of 47 cases with emphasis on the value of electron microscopy. Ultrastruct Pathol. 2014;38(6):367–76. 25191813 10.3109/01913123.2014.952803

[23] Ghembaza A , Boleto G , Bommelaer M , Karras A , Javaugue V , Bridoux F , et al. Prognosis and long‐term outcomes in type I cryoglobulinemia: a multicenter study of 168 patients. Am J Hematol. 2023;98(7):1080–6. 37139676 10.1002/ajh.26944

[24] Terrier B , Karras A , Kahn JE , Le Guenno G , Marie I , Benarous L , et al. The spectrum of type I cryoglobulinemia vasculitis: new insights based on 64 cases. Medicine (Baltimore). 2013;92(2):61–8. 23429354 10.1097/MD.0b013e318288925c PMC4553985

[25] Harel S , Mohr M , Jahn I , Aucouturier F , Galicier L , Asli B , et al. Clinico‐biological characteristics and treatment of type I monoclonal cryoglobulinaemia: a study of 64 cases. Br J Haematol. 2015;168(5):671–8. 25363150 10.1111/bjh.13196

[26] Retamozo S , Díaz‐Lagares C , Bosch X , Bové A , Brito‐Zerón P , Gómez ME , et al. Life‐threatening cryoglobulinemic patients with hepatitis C: clinical description and outcome of 279 patients. Medicine (Baltimore). 2013;92(5):273–84. 23974248 10.1097/MD.0b013e3182a5cf71 PMC4553974

[27] Ferri C , Greco F , Longombardo G , Palla P , Moretti A , Marzo E , et al. Antibodies to hepatitis C virus in patients with mixed cryoglobulinemia. Arthritis Rheum. 1991;34(12):1606–10. 1660716 10.1002/art.1780341221

[28] Zignego AL , Ferri C , Giannini C , La Civita L , Careccia G , Longombardo G , et al. Hepatitis C virus infection in mixed cryoglobulinemia and B‐cell non‐Hodgkin's lymphoma: evidence for a pathogenetic role. Arch Virol. 1997;142(3):545–55. 9349300 10.1007/s007050050100

[29] Casato M , Taliani G , Pucillo LP , Goffredo F , Laganà B , Bonomo L . Cryoglobulinaemia and hepatitis C virus. Lancet. 1991;337(8748):1047–8. 10.1016/0140-6736(91)92715-e 1673206

[30] Zignego AL , Ramos‐Casals M , Ferri C , Saadoun D , Arcaini L , Roccatello D , et al. International therapeutic guidelines for patients with HCV‐related extrahepatic disorders. A multidisciplinary expert statement. Autoimmun Rev. 2017;16(5):523–41. 28286108 10.1016/j.autrev.2017.03.004

[31] Zignego AL , Bréchot C . Extrahepatic manifestations of HCV infection: facts and controversies. J Hepatol. 1999;31(2):369–76. 10453955 10.1016/s0168-8278(99)80239-6

[32] Zignego AL , Ferri C , Pileri SA , Caini P , Bianchi FB , infection IAotSoLCoEMoH. Extrahepatic manifestations of hepatitis C Virus infection: a general overview and guidelines for a clinical approach. Dig Liver Dis. 2007;39(1):2–17. 16884964 10.1016/j.dld.2006.06.008

[33] Ferri C , Zignego AL , Antonelli A . Extrahepatic manifestations of chronic HCV infection. N Engl J Med. 2021;385(1):94. 10.1056/NEJMc2106143 34192442

[34] Ferri C , La Civita L , Longombardo G , Zignego AL , Pasero G . Mixed cryoglobulinaemia: a cross‐road between autoimmune and lymphoproliferative disorders. Lupus. 1998;7(4):275–9. 9643318 10.1191/096120398678920091

[35] Ferri C , Antonelli A , Mascia MT , Sebastiani M , Fallahi P , Ferrari D , et al. HCV‐related autoimmune and neoplastic disorders: the HCV syndrome. Dig Liver Dis. 2007;39 (Suppl 1):S13–21. 17936215 10.1016/s1590-8658(07)80005-3

[36] Ferri C , Sebastiani M , Giuggioli D , Cazzato M , Longombardo G , Antonelli A , et al. Mixed cryoglobulinemia: demographic, clinical, and serologic features and survival in 231 patients. Semin Arthritis Rheum. 2004;33(6):355–74. 15190522 10.1016/j.semarthrit.2003.10.001

[37] Bernacchi E , Civita LL , Caproni M , Zignego AL , Bianchi B , Monti M , et al. Hepatitis C virus (HCV) in cryoglobulinaemic leukocytoclastic vasculitis (LCV): could the presence of HCV in skin lesions be related to T CD8+ lymphocytes, HLA‐DR and ICAM‐1 expression? Exp Dermatol. 1999;8(6):480–6. 10597137 10.1111/j.1600-0625.1999.tb00306.x

[38] La Civita L , Zignego AL , Lombardini F , Monti M , Longombardo G , Pasero G , et al. Exacerbation of peripheral neuropathy during alpha‐interferon therapy in a patient with mixed cryoglobulinemia and hepatitis B virus infection. J Rheumatol. 1996;23(9):1641–3. 8877939

[39] Pasquariello A , Ferri C , Moriconi L , La Civita L , Longombardo G , Lombardini F , et al. Cryoglobulinemic membranoproliferative glomerulonephritis associated with hepatitis C virus. Am J Nephrol. 1993;13(4):300–4. 7505528 10.1159/000168641

[40] Pietrogrande M , De Vita S , Zignego AL , Pioltelli P , Sansonno D , Sollima S , et al. Recommendations for the management of mixed cryoglobulinemia syndrome in hepatitis C virus‐infected patients. Autoimmun Rev. 2011;10(8):444–54. 21303705 10.1016/j.autrev.2011.01.008

[41] Jorgensen C , Legouffe MC , Perney P , Coste J , Tissot B , Segarra C , et al. Sicca syndrome associated with hepatitis C virus infection. Arthritis Rheum. 1996;39(7):1166–71. 8670326 10.1002/art.1780390714

[42] Quartuccio L , Petrarca A , Mansutti E , Pieroni S , Calcabrini L , Avellini C , et al. Efficacy of rituximab in severe and mild abdominal vasculitis in the course of mixed cryoglobulinemia. Clin Exp Rheumatol. 2010;28(1 Suppl 57):84–7. 20412709

[43] Terrier B , Saadoun D , Sène D , Scerra S , Musset L , Cacoub P . Presentation and outcome of gastrointestinal involvement in hepatitis C virus‐related systemic vasculitis: a case‐control study from a single‐centre cohort of 163 patients. Gut. 2010;59(12):1709–15. 20841367 10.1136/gut.2010.218123

[44] Ferri C , La Civita L , Fazzi P , Solfanelli S , Lombardini F , Begliomini E , et al. Interstitial lung fibrosis and rheumatic disorders in patients with hepatitis C virus infection. Br J Rheumatol. 1997;36(3):360–5. 9133969 10.1093/rheumatology/36.3.360

[45] Mazzaro C , Dal Maso L , Mauro E , Visentini M , Tonizzo M , Gattei V , et al. Hepatitis C virus‐related cryoglobulinemic vasculitis: a review of the role of the new direct antiviral agents (DAAs) therapy. Autoimmun Rev. 2020;19(8):102589. 32540448 10.1016/j.autrev.2020.102589

[46] Ferri C , Caracciolo F , Zignego AL , La Civita L , Monti M , Longombardo G , et al. Hepatitis C virus infection in patients with non‐Hodgkin's lymphoma. Br J Haematol. 1994;88(2):392–4. 7803287 10.1111/j.1365-2141.1994.tb05036.x

[47] Ferri C , La Civita L , Caracciolo F , Zignego AL . Non‐Hodgkin's lymphoma: possible role of hepatitis C virus. JAMA. 1994;272(5):355–6. 10.1001/jama.1994.03520050033023 8028163

[48] Fallahi P , Ferrari SM , Ruffilli I , Elia G , Giuggioli D , Colaci M , et al. Incidence of thyroid disorders in mixed cryoglobulinemia: results from a longitudinal follow‐up. Autoimmun Rev. 2016;15(7):747–51. 26970485 10.1016/j.autrev.2016.03.012

[49] Desbois AC , Cacoub P . Diabetes mellitus, insulin resistance and hepatitis C virus infection: a contemporary review. World J Gastroenterol. 2017;23(9):1697–711. 28321170 10.3748/wjg.v23.i9.1697 PMC5340821

[50] Antonelli A , Ferri C , Fallahi P , Sebastiani M , Nesti C , Barani L , et al. Type 2 diabetes in hepatitis C‐related mixed cryoglobulinaemia patients. Rheumatology (Oxford). 2004;43(2):238–40. 13130149 10.1093/rheumatology/keh011

[51] Levo Y , Gorevic PD , Kassab HJ , Tobias H , Franklin EC . Liver involvement in the syndrome of mixed cryoglobulinemia. Ann Intern Med. 1977;87(3):287–92. 900672 10.7326/0003-4819-87-3-287

[52] Monti G , Saccardo F , Pioltelli P , Rinaldi G . The natural history of cryoglobulinemia: symptoms at onset and during follow‐up. A report by the Italian group for the study of cryoglobulinemias (GISC). Clin Exp Rheumatol. 1995; Suppl 13: S129–33. 8730493

[53] Terrier B , Marie I , Lacraz A , Belenotti P , Bonnet F , Chiche L , et al. Non HCV‐related infectious cryoglobulinemia vasculitis: results from the French nationwide CryoVas survey and systematic review of the literature. J Autoimmun. 2015;65:74–81. 26320984 10.1016/j.jaut.2015.08.008

[54] Mazzaro C , Dal Maso L , Mauro E , Gattei V , Ghersetti M , Bulian P , et al. Survival and prognostic factors in mixed cryoglobulinemia: data from 246 cases. Diseases. 2018;6(2):35. 29751499 10.3390/diseases6020035 PMC6023473

[55] Bai W , Zhang L , Zhao J , Zhang S , Zhou J , Leng X , et al. Renal involvement and HBV infection are common in Chinese patients with cryoglobulinemia. Front Immunol. 2021;12:580271. 33717064 10.3389/fimmu.2021.580271 PMC7947000

[56] Li SJ , Xu ST , Chen HP , Zhang MC , Xu F , Cheng SQ , et al. Clinical and morphologic spectrum of renal involvement in patients with HBV‐associated cryoglobulinaemia. Nephrology (Carlton). 2017;22(6):449–55. 27062412 10.1111/nep.12795

[57] Cacoub P , Asselah T . Hepatitis B virus infection and extra‐hepatic manifestations: a systemic disease. Am J Gastroenterol. 2022;117(2):253–63. 34913875 10.14309/ajg.0000000000001575

[58] Boglione L , D'Avolio A , Cariti G , Di Perri G . Telbivudine in the treatment of hepatitis B‐associated cryoglobulinemia. J Clin Virol. 2013;56(2):167–9. 23182457 10.1016/j.jcv.2012.10.014

[59] Mazzaro C , Dal Maso L , Urraro T , Mauro E , Castelnovo L , Casarin P , et al. Hepatitis B virus related cryoglobulinemic vasculitis: a multicentre open label study from the Gruppo Italiano di Studio delle Crioglobulinemie‐GISC. Dig Liver Dis. 2016;48(7):780–4. 27106525 10.1016/j.dld.2016.03.018

[60] Zignego AL , Macchia D , Monti M , Thiers V , Mazzetti M , Foschi M , et al. Infection of peripheral mononuclear blood cells by hepatitis C virus. J Hepatol. 1992;15(3):382–6. 1332999 10.1016/0168-8278(92)90073-x

[61] Gragnani L , Lorini S , Marri S , Basile U , Santarlasci V , Monti M , et al. Hematological and genetic markers in the rational approach to patients with HCV sustained virological response with or without persisting cryoglobulinemic vasculitis. Hepatology. 2021;74(3):1164–1173. 33721342 10.1002/hep.31804 PMC8519006

[62] Ferri C , Sebastiani M , Giuggioli D , Colaci M , Fallahi P , Piluso A , et al. Hepatitis C virus syndrome: a constellation of organ‐ and non‐organ specific autoimmune disorders, B‐cell non‐Hodgkin's lymphoma, and cancer. World J Hepatol. 2015;7(3):327–43. 25848462 10.4254/wjh.v7.i3.327 PMC4381161

[63] Giannini C , Gragnani L , Piluso A , Caini P , Petrarca A , Monti M , et al. Can BAFF promoter polymorphism be a predisposing condition for HCV‐related mixed cryoglobulinemia? Blood. 2008;112(10):4353–4. 18988879 10.1182/blood-2008-07-170613

[64] Lake‐Bakaar G , Jacobson I , Talal A . B cell activating factor (BAFF) in the natural history of chronic hepatitis C virus liver disease and mixed cryoglobulinaemia. Clin Exp Immunol. 2012;170(2):231–7. 23039894 10.1111/j.1365-2249.2012.04653.x PMC3482370

[65] Gragnani L , Lorini S , Marri S , Rattotti S , Madia F , Zibellini S , et al. B‐cell activating factor (BAFF), BAFF promoter and BAFF receptor allelic variants in hepatitis C virus related cryoglobulinemic vasculitis and non‐Hodgkin's lymphoma. Hematol Oncol. 2022;40(4):658–66. 35460540 10.1002/hon.3008 PMC9790294

[66] Machida K , Cheng KT , Sung VM , Shimodaira S , Lindsay KL , Levine AM , et al. Hepatitis C virus induces a mutator phenotype: enhanced mutations of immunoglobulin and protooncogenes. Proc Natl Acad Sci USA. 2004;101(12):4262–7. 14999097 10.1073/pnas.0303971101 PMC384729

[67] Zignego AL , Giannini C , Monti M , Gragnani L . Hepatitis C virus lymphotropism: lessons from a decade of studies. Dig Liver Dis. 2007; 39(Suppl 1):S38–45. 17936221 10.1016/s1590-8658(07)80009-0

[68] Young C , Singh M , Jackson KJL , Field MA , Peters TJ , Angioletti‐Uberti S , et al. A triad of somatic mutagenesis converges in self‐reactive B cells to cause a virus‐induced autoimmune disease. Immunity. 2025;58(2):412–30.e10. 39818208 10.1016/j.immuni.2024.12.011

[69] Visentini M , Cagliuso M , Conti V , Carbonari M , Casato M , Fiorilli M . The V(H)1–69‐expressing marginal zone B cells expanded in HCV‐associated mixed cryoglobulinemia display proliferative anergy irrespective of CD21(low) phenotype. Blood. 2011;118(12):3440–1. 21940829 10.1182/blood-2011-05-353821

[70] Charles ED , Green RM , Marukian S , Talal AH , Lake‐Bakaar GV , Jacobson IM , et al. Clonal expansion of immunoglobulin M^+^CD27^+^ B cells in HCV‐associated mixed cryoglobulinemia. Blood. 2008;111(3):1344–56. 17942751 10.1182/blood-2007-07-101717 PMC2214737

[71] Comarmond C , Lorin V , Marques C , Maciejewski‐Duval A , Joher N , Planchais C , et al. TLR9 signalling in HCV‐associated atypical memory B cells triggers Th1 and rheumatoid factor autoantibody responses. J Hepatol. 2019;71(5):908–19. 31279905 10.1016/j.jhep.2019.06.029

[72] Visentini M , Cagliuso M , Conti V , Carbonari M , Cibati M , Siciliano G , et al. Clonal B cells of HCV‐associated mixed cryoglobulinemia patients contain exhausted marginal zone‐like and CD21 low cells overexpressing Stra13. Eur J Immunol. 2012;42(6):1468–76. 22678901 10.1002/eji.201142313

[73] Del Padre M , Marrapodi R , Minafò YA , Piano Mortari E , Radicchio G , Bocci C , et al. Dual stimulation by autoantigen and CpG fosters the proliferation of exhausted rheumatoid factor‐specific CD21. Front Immunol. 2023;14:1094871. 36845129 10.3389/fimmu.2023.1094871 PMC9945227

[74] Visentini M , Gragnani L , Santini SA , Urraro T , Villa A , Monti M , et al. Flares of mixed cryoglobulinaemia vasculitis after vaccination against SARS‐CoV‐2. Ann Rheum Dis. 2022;81(3):441–3. 34819272 10.1136/annrheumdis-2021-221248

[75] Gragnani L , Fognani E , Piluso A , Zignego AL . Hepatitis C virus‐related mixed cryoglobulinemia: is genetics to blame? World J Gastroenterol. 2013;19(47):8910–5. 24379615 10.3748/wjg.v19.i47.8910 PMC3870543

[76] Couronné L , Bachy E , Roulland S , Nadel B , Davi F , Armand M , et al. From hepatitis C virus infection to B‐cell lymphoma. Ann Oncol. 2018;29(1):92–100. 29045541 10.1093/annonc/mdx635

[77] Rieu V , Cohen P , André MH , Mouthon L , Godmer P , Jarrousse B , et al. Characteristics and outcome of 49 patients with symptomatic cryoglobulinaemia. Rheumatology (Oxford). 2002;41(3):290–300. 11934966 10.1093/rheumatology/41.3.290

[78] Payet J , Livartowski J , Kavian N , Chandesris O , Dupin N , Wallet N , et al. Type I cryoglobulinemia in multiple myeloma, a rare entity: analysis of clinical and biological characteristics of seven cases and review of the literature. Leuk Lymphoma. 2013;54(4):767–77. 22385269 10.3109/10428194.2012.671481

[79] Néel A , Perrin F , Decaux O , Dejoie T , Tessoulin B , Halliez M , et al. Long‐term outcome of monoclonal (type 1) cryoglobulinemia. Am J Hematol. 2014;89(2):156–61. 24532335 10.1002/ajh.23608

[80] Sidana S , Rajkumar SV , Dispenzieri A , Lacy MQ , Gertz MA , Buadi FK , et al. Clinical presentation and outcomes of patients with type 1 monoclonal cryoglobulinemia. Am J Hematol. 2017;92(7):668–73. 28370486 10.1002/ajh.24745 PMC5579826

[81] Zhang L , Zhang W , Zhou D . Long‐term treatment response to PD‐1 blockade therapy in a patient with DLBCL relapsed after anti‐CD19 chimeric antigen receptor T cell treatment. Ann Hematol. 2021;100(1):289–91. 32335701 10.1007/s00277-020-03993-9

[82] Zignego AL , Pawlotsky JM , Bondin M , Cacoub P . Expert opinion on managing chronic HCV in patients with mixed cryoglobulinaemia vasculitis. Antivir Ther. 2018;23(Suppl 2):1–9. 10.3851/IMP3246 30451151

[83] Ramos‐Casals M , Zignego AL , Ferri C , Brito‐Zerón P , Retamozo S , Casato M , et al. Evidence‐based recommendations on the management of extrahepatic manifestations of chronic hepatitis C virus infection. J Hepatol. 2017;66(6):1282–99. 28219772 10.1016/j.jhep.2017.02.010

[84] Ferri C , Sebastiani M , Saadoun D , Cacoub P . EULAR compendium on rheumatic diseases. In: Bijsma. EJ , editor. EULAR compendium on rheumatic diseases: London, BMJ. Annals of the Rheumatic Diseases. Publishing Group Ltd; 2012. p. 1042–71.

[85] Gragnani L , Visentini M , Lorini S , Santini SA , Lauletta G , Mazzaro C , et al. COVID‐19 and mixed cryoglobulinemia syndrome: long‐term survey study on the prevalence and outcome, vaccine safety, and immunogenicity. J Clin Immunol. 2023;43(4):680–91. 36795264 10.1007/s10875-023-01444-4 PMC9933006

[86] Visentini M , Quartuccio L , Del Padre M , Colantuono S , Minafò YA , Fiorilli M , et al. Late relapses of hepatitis C virus‐cured mixed cryoglobulinaemia associated with infection or cancer. Rheumatology (Oxford). 2018;57(10):1870–1. 29868896 10.1093/rheumatology/key157

[87] Bonacci M , Lens S , Mariño Z , Londoño MC , Rodriguez‐Tajes S , Sánchez‐Tapias JM , et al. Long‐term outcomes of patients with HCV‐associated cryoglobulinemic vasculitis after virologic cure. Gastroenterology. 2018;155(2):311–5.e6. 29705529 10.1053/j.gastro.2018.04.024

[88] Gragnani L , Cerretelli G , Lorini S , Steidl C , Giovannelli A , Monti M , et al. Interferon‐free therapy in hepatitis C virus mixed cryoglobulinaemia: a prospective, controlled, clinical and quality of life analysis. Aliment Pharmacol Ther. 2018;48(4):440–50. 29952013 10.1111/apt.14845

[89] Gragnani L , Visentini M , Fognani E , Urraro T , De Santis A , Petraccia L , et al. Prospective study of guideline‐tailored therapy with direct‐acting antivirals for hepatitis C virus‐associated mixed cryoglobulinemia. Hepatology. 2016;64(5):1473–82. 27483451 10.1002/hep.28753

[90] Gragnani L , Piluso A , Urraro T , Fabbrizzi A , Fognani E , Petraccia L , et al. Virological and clinical response to interferon‐free regimens in patients with HCV‐related mixed cryoglobulinemia: preliminary results of a prospective pilot study. Curr Drug Targets. 2017;18(7):772–85. 26853322 10.2174/1389450117666160208145432

[91] Kondili LA , Monti M , Quaranta MG , Gragnani L , Panetta V , Brancaccio G , et al. A prospective study of direct‐acting antiviral effectiveness and relapse risk in HCV cryoglobulinemic vasculitis by the Italian PITER cohort. Hepatology. 2022;76(1):220–32. 34919289 10.1002/hep.32281 PMC9305531

[92] Reiberger T , Lens S , Cabibbo G , Nahon P , Zignego AL , Deterding K , et al. EASL position paper on clinical follow‐up after HCV cure. J Hepatol. 2024;81(2):326–44. 38845253 10.1016/j.jhep.2024.04.007

[93] Pozzato G , Mazzaro C , Artemova M , Abdurakhmanov D , Grassi G , Crosato I , et al. Direct‐acting antiviral agents for hepatitis C virus‐mixed cryoglobulinaemia: dissociated virological and haematological responses. Br J Haematol. 2020;191(5):775–783. 32790920 10.1111/bjh.17036

[94] Zignego AL , Ferri C , Giannelli F , Giannini C , Caini P , Monti M , et al. Prevalence of bcl‐2 rearrangement in patients with hepatitis C virus‐related mixed cryoglobulinemia with or without B‐cell lymphomas. Ann Intern Med. 2002;137(7):571–80. 12353944 10.7326/0003-4819-137-7-200210010-00008

[95] Giannelli F , Moscarella S , Giannini C , Caini P , Monti M , Gragnani L , et al. Effect of antiviral treatment in patients with chronic HCV infection and *t*(14;18) translocation. Blood. 2003;102(4):1196–201. 12689948 10.1182/blood-2002-05-1537

[96] Cacoub P , Saadoun D . Extrahepatic manifestations of chronic HCV infection. N Engl J Med. 2021;384(11):1038–52. 33730456 10.1056/NEJMra2033539

[97] Mazzaro C , Visentini M , Gragnani L , Vit F , Tissino E , Pozzo F , et al. Persistence of monoclonal B‐cell expansion and intraclonal diversification despite virus eradication in patients affected by hepatitis C virus‐associated lymphoproliferative disorders. Br J Haematol. 2023;203(2):237–43. 37491625 10.1111/bjh.19002

[98] Quartuccio L , Bortoluzzi A , Scirè CA , Marangoni A , Del Frate G , Treppo E , et al. Management of mixed cryoglobulinemia with rituximab: evidence and consensus‐based recommendations from the Italian study group of cryoglobulinemia (GISC). Clin Rheumatol. 2023;42(2):359–70. 36169798 10.1007/s10067-022-06391-w PMC9873783

[99] Petrarca A , Rigacci L , Monti M , Giannini C , Bernardi F , Caini P , et al. Improvement in liver cirrhosis after treatment of HCV‐related mixed cryoglobulinemia with rituximab. Dig Liver Dis. 2007;39(Suppl 1):S129–33. 17936214 10.1016/s1590-8658(07)80025-9

[100] Visentini M , Ludovisi S , Petrarca A , Pulvirenti F , Zaramella M , Monti M , et al. A phase II, single‐arm multicenter study of low‐dose rituximab for refractory mixed cryoglobulinemia secondary to hepatitis C virus infection. Autoimmun Rev. 2011;10(11):714–9. 21570494 10.1016/j.autrev.2011.04.033

[101] De Vita S , Quartuccio L , Isola M , Mazzaro C , Scaini P , Lenzi M , et al. A randomized controlled trial of rituximab for the treatment of severe cryoglobulinemic vasculitis. Arthritis Rheum. 2012;64(3):843–53. 22147661 10.1002/art.34331

[102] Quartuccio L , Zuliani F , Corazza L , Scaini P , Zani R , Lenzi M , et al. Retreatment regimen of rituximab monotherapy given at the relapse of severe HCV‐related cryoglobulinemic vasculitis: long‐term follow up data of a randomized controlled multicentre study. J Autoimmun. 2015;63:88–93. 26255249 10.1016/j.jaut.2015.07.012

[103] Desbois AC , Biard L , Sène D , Brocheriou I , Rouvier P , Lioger B , et al. Rituximab‐associated vasculitis flare: incidence, predictors, and outcome. J Rheumatol. 2020;47(6):896–902. 31371658 10.3899/jrheum.190076

[104] Marjot T , Eberhardt CS , Boettler T , Belli LS , Berenguer M , Buti M , et al. Impact of COVID‐19 on the liver and on the care of patients with chronic liver disease, hepatobiliary cancer, and liver transplantation: an updated EASL position paper. J Hepatol. 2022;77(4):1161–97. 35868584 10.1016/j.jhep.2022.07.008 PMC9296253

[105] Gragnani L , Lorini S , Marri S , Vacchi C , Madia F , Monti M , et al. Predictors of long‐term cryoglobulinemic vasculitis outcomes after HCV eradication with direct‐acting antivirals in the real‐life. Autoimmun Rev. 2022;21(1):102923. 34419670 10.1016/j.autrev.2021.102923

[106] Basile U , Gragnani L , Piluso A , Gulli F , Urraro T , Dell'Abate MT , et al. Assessment of free light chains in HCV‐positive patients with mixed cryoglobulinaemia vasculitis undergoing rituximab treatment. Liver Int. 2015;35(9):2100–7. 25800731 10.1111/liv.12829

[107] Basile U , Gulli F , Gragnani L , Napodano C , Pocino K , Rapaccini GL , et al. Free light chains: eclectic multipurpose biomarker. J Immunol Methods. 2017;451:11–9. 28931470 10.1016/j.jim.2017.09.005

[108] Fayed A , Hegazy MT , Biard L , Vieira M , El Shabony T , Saadoun D , et al. Relapse of hepatitis C virus cryoglobulinemic vasculitis after sustained viral response after interferon‐free direct‐acting antivirals. Am J Gastroenterol. 2022;117(4):627–36. 35103020 10.14309/ajg.0000000000001667

[109] Han HX , Su W , Zhou DB , Li J , Cao XX . Hepatitis B virus‐related cryoglobulinemia: clinical characteristics, virological features, and treatment. Virus Res. 2023;336:199212. 37640269 10.1016/j.virusres.2023.199212 PMC10474225

[110] Mazzaro C , Bomben R , Picco L , Zanier A , Schioppa O , Gattei V . Relapse of mixed cryoglobulinemia despite sustained virologic response with nucleotide analogues therapy in chronic hepatitis B. Rheumatology (Oxford). 2025;64(7):4422–4424. 40053695 10.1093/rheumatology/keaf042

[111] Galli M , Oreni L , Saccardo F , Castelnovo L , Filippini D , Marson P , et al. HCV‐unrelated cryoglobulinaemic vasculitis: the results of a prospective observational study by the Italian group for the study of cryoglobulinaemias (GISC). Clin Exp Rheumatol. 2017;35(Suppl 103(1)):67–76. 28466806

[112] Terrier B , Krastinova E , Marie I , Launay D , Lacraz A , Belenotti P , et al. Management of noninfectious mixed cryoglobulinemia vasculitis: data from 242 cases included in the CryoVas survey. Blood. 2012;119(25):5996–6004. 22474249 10.1182/blood-2011-12-396028

[113] Micheletti RG , Pagnoux C . Management of cutaneous vasculitis. Presse Med. 2020;49(3):104033. 32645416 10.1016/j.lpm.2020.104033

[114] Lauletta G , Russi S , Pavone F , Vacca A , Dammacco F . Direct‐acting antiviral agents in the therapy of hepatitis C virus‐related mixed cryoglobulinaemia: a single‐centre experience. Arthritis Res Ther. 2017;19(1):74. 28388935 10.1186/s13075-017-1280-6 PMC5385046

[115] Emery JS , Kuczynski M , La D , Almarzooqi S , Kowgier M , Shah H , et al. Efficacy and safety of direct acting antivirals for the treatment of mixed cryoglobulinemia. Am J Gastroenterol. 2017;112(8):1298–308. 28291241 10.1038/ajg.2017.49

[116] Mazzaro C , Dal Maso L , Quartuccio L , Ghersetti M , Lenzi M , Mauro E , et al. Long‐term effects of the new direct antiviral agents (DAAs) therapy for HCV‐related mixed cryoglobulinaemia without renal involvement: a multicentre open‐label study. Clin Exp Rheumatol. 2018;36(Suppl 111(2)):107–14. 29465371

[117] Passerini M , Schiavini M , Magni CF , Landonio S , Niero F , Passerini S , et al. Are direct‐acting antivirals safe and effective in hepatitis C virus‐cryoglobulinemia? virological, immunological, and clinical data from a real‐life experience. Eur J Gastroenterol Hepatol. 2018;30(10):1208–15. 30138160 10.1097/MEG.0000000000001239

[118] Cacoub P , Si Ahmed SN , Ferfar Y , Pol S , Thabut D , Hezode C , et al. Long‐term efficacy of interferon‐free antiviral treatment regimens in patients with hepatitis C virus‐associated cryoglobulinemia vasculitis. Clin Gastroenterol Hepatol. 2019;17(3):518–26. 29857143 10.1016/j.cgh.2018.05.021

